# Supplementary material for: Dynamic risk assessment of hospital oxygen supply system by HAZOP and intuitionistic fuzzy
Source: PLoS One. 2023 Feb 23;18(2):e0280918. doi: 10.1371/journal.pone.0280918 (PMC9949673; doi:10.1371/journal.pone.0280918)
Supplement: Attachment — Submitted filename: Review to PLOSE one.pdf [file pone.0280918.s002.pdf]

Review report to PLOSE ONE

Review on research titled" Dynamic risk assessment of hospital oxygen supply system…"

General comments:

Title: your title "Dynamic risk assessment of hospital oxygen supply system by pressure swing absorbance technology based on the integration of HAZOP technique and intuitionistic fuzzy set" is too long. Make it short and precise, maximum of 10-15 words.

I suggest "Dynamic risk assessment of hospital oxygen supply system" or "Dynamic risk assessment of hospital oxygen supply system by pressure swing absorbance technology" and Add where and when the research was done.

Abstract:

Background: The background of information of your abstract is too extended. Try to minimize it.

Methods: where and when you are assessing; is your research is qualitative or quantitative? Incorporate such information.

Result: what are the most frequent deviations you identified by HAZOP tool?

Introduction

Good introduction but your paragraphs (e.g. 1st paragraph) are too bulky and different issues are included in a single paragraph. So make your paragraph specific.

Result:

1. write all 9 deviation you found and report the with high magnitude as table 4
2. Your line 376 saying" The results showed some differences between the findings of both approaches, **which can be seen in**…. remove such expressions and just put (**Table 6**).

Suggestion: try to incorporate the above comments

Decision: Accepted with minor modifications

Revirewer: Mr Asamirew Alemu (BSc, MSc), Mizan-Tepi University, Ethiopia.
