# Supplementary material for: Dynamic risk assessment of hospital oxygen supply system by HAZOP and intuitionistic fuzzy
Source: PLoS One. 2023 Feb 23;18(2):e0280918. doi: 10.1371/journal.pone.0280918 (PMC9949673; doi:10.1371/journal.pone.0280918)
Supplement: Attachment — Submitted filename: dr. hazim alhiti Review Report 542.doc [file pone.0280918.s001.doc]

**This is the review report of Dr. Hazim Abdul Rahman Alhiti**

**I The Information of** Research Paper Review

| **Paper’s File Name** | PONE-D-22-22776 | | | | |
| --- | --- | --- | --- | --- | --- |
| **Journal Title** | PLOS ONE | | | | |
| **Paper Title** | Dynamic risk assessment of hospital oxygen supply system by pressure swing  absorbance technology based on the integration of HAZOP technique and intuitionistic  fuzzy set | | | | |
| **Date received** | 20/5/2022 | | | | |
| **Date reviewed** | 25/5/2022 | | | | |
| **Reviewer’s Name** | **HAZIM ABDUL RAHMAN ALHITI** | | | | |
| **Affiliation** | **General Surgeon Specialist M.D**  **Al-Ramadi Teaching Hospital**  **Chief of Breast Clinic (Hit General Hospital)-Iraq**  **Chief of cancer Clinic (Hit General Hospital)-Iraq**  **Academic lecturer in college of medicine- Fallujah University**  **Academic external examiner- college of medicine-University of Al-Qadisiyah**  **Academic lecturer in Higher Institute of Health, Al Anbar Directorate of Health**  **Head of Emergency Faculty in higher Institute of health,**  **Academic Lecturer in higher Institute of health,**  **Al Anbar Directorate of Health-Iraq**  **Author, Reviewer, Editor, Book writer.**  [**hazim.alhiti@uoanbar.edu.iq**](mailto:hazim.alhiti@uoanbar.edu.iq)  [**hazim.abdulrahman@uofallujah.edu.iq**](mailto:hazim.abdulrahman@uofallujah.edu.iq)  [**Hazim4436@gmail.com**](mailto:Hazim4436@gmail.com)  [**https://publons.com/researcher/1830217/hazim-abdul-rahman-alhiti/**](https://publons.com/researcher/1830217/hazim-abdul-rahman-alhiti/)  [**https://orcid.org/0000-0003-0000-8267**](https://orcid.org/0000-0003-0000-8267)  [**https://scholar.google.com/citations?user=GeT21MkAAAAJ&hl=ar&authuser=2**](https://scholar.google.com/citations?user=GeT21MkAAAAJ&hl=ar&authuser=2)  [**https://www.researchgate.net/profile/Hazim-Alhiti**](https://www.researchgate.net/profile/Hazim-Alhiti)  [**https://iraqiboard.academia.edu/TheHazim**](https://iraqiboard.academia.edu/TheHazim) | | | | |
| **General comments** | | | | | |
| 1. **the main content of research presented in the paper is Dynamic risk assessment of hospital oxygen supply system by pressure swing absorbance technology based on the integration of HAZOP technique and intuitionistic fuzzy set** 2. **the main proposed paper is to quantitatively identify the significant deviations of the oxygen supply system as critical equipment at hospitals.** 3. **the deduced conclusions based on the research methods/cases are needs assessment tool to optimize** 4. **maintenance programs and provide the necessary training for the staff, maintenance operators, and medical equipment managers.** 5. **the conclusions are tenable** 6. **the progress of this paper is good compared with the current research results** | | | | | |
| **Strengths and weaknesses** | | | | | |
| 1. **the topic is not unique, but worthy of researching** 2. **there are hundreds of papers in google scholar about this topic since 2018** 3. **the title is too long. Please shorten it to be attractive and citable** 4. **the abstract is too long. Please shorten it and revise the plagiarism** 5. **the aim is clear** 6. **the KEYWORDS are good.** 7. **lack of the abbreviations section** 8. **the introduction provide sufficient background information for readers in the immediate field to understand the problem/hypotheses** 9. **the text arrangement is good** 10. **the logic is clear** 11. **the paper is not novel** 12. **there are few grammatical errors in this article** 13. **the related concepts are introduced** 14. **the readability is sufficient** 15. **the results are good** 16. **all figures/tables are clear enough to summarize the results for presentation to the readers** 17. **all figures/tables are well referred to in the text** 18. **the theoretical analysis in this article is sufficient** 19. **the discussion of results from multiple angles is sufficient** 20. **the conclusion are good** 21. **the reference section are good** 22. **the references are in order within the text** 23. **Bias is acceptable** 24. **Fund is mentioned** 25. **Ethical approval is not needed** 26. **Conflict of interest** **is mentioned** 27. **Acknowledgement** **is mentioned** 28. **You can use my suggestions**   **My final decision is acceptable after minor revision** | | | | | |
| **Paper score** | | | | | |
| **Ten-point System** | **Relevance** | **Originality** | **Significance** | **Technical soundness** | **Clarity of presentation / language** |
| **Enter a score between 0** **to 10** | **7** | **9** | **9** | **9** | **9** |
| **Final Evaluation** | | | | | |
| **☐ Excellent** **☐ Very Good** **☐ Good** **☐ Fair** **☐ Poor** | | | | | |
| **☐ Acceptance** **☐ Minor Revision** **☐ Major Revision** **☐ Rejection** | | | | | |

| **Part No.** | **Description of the Part of Manuscript** | **Score**  **0 to 10** |
| --- | --- | --- |
| 1. | **Title**  a. Accurate | 9 |
|  | b. Effective | 5 |
|  | c. Represents Main Message of Paper | 9 |
| 2. | **ABSTRACT**  a. Size of abstract (maximum 250 words) | 4 |
|  | b. Objective | 9 |
|  | c. Methodology | 9 |
|  | d. Results | 9 |
|  | e. Conclusion | 9 |
|  | f. Key Words | 9 |
| 3. | difficult words/key words if relevant | 5 |
| 4. | **introduction**  a. It start Relevant | 9 |
|  | b. Subject of Paper Made Clear | 9 |
|  | c. Importance of Study Made Clear | 9 |
|  | d. Reasons for the Study Stated | 9 |
|  | e. Significant finding of the Study Stated | 9 |
|  | f. Available International, National, Ancient Literature Recorded with  References of at least 05 years (60%) and in Vancouver style | 7 |
|  | g. References appropriate for Discussion are not to be mentioned in  Introduction | 7 |
| 5 | **Aims and objectives of the study**  a. Are clearly Stated | 9 |
|  | b. Are relevant with study | 9 |
| 6 | **methodology**  a. Population of uniform standard | 9 |
|  | b. Sample selection criteria reliable | 9 |
|  | c. Inclusion/Exclusion criteria specified | 9 |
|  | d. Control within the study or literature given | - |
|  | e. Important Variable Measurement Criteria specified | 9 |
|  | f. Investigation/Procedure/Technique clearly given | 9 |
| 7 | **Results and analysis of results**  a. Presentation of results clear | 9 |
|  | b. Data relevant to objectives of the study | 9 |
|  | c. All subjects/Variable in the study accounted for | 9 |
|  | d. Results reported the drop out (if present) | 9 |
|  | e. Results presented in appropriately | 9 |
|  | f. Results presented with standard deviation | - |
|  | g. Appropriate statistical tests applied | - |
|  | h. Data Analysis correctly interpreted | 0 |
|  | i. Level of significance (preferred less than 0.1%) | - |
| 8 | **Discussion**  a. Important findings and results discussed | 9 |
|  | b. New emerging findings discussed | 9 |
|  | c. Results of the study compared/discussed with other past studies | 9 |
|  | d. Results and analysis leads to firm conclusions | 9 |
| 9 | **Conclusions**  a. Conclusion is in accordance with data analysis and results | 9 |
|  | b. Conclusions relevant to the aims and objectives of the study | 9 |
|  | c. Conclusions based on critical argument | 9 |
|  | d. Future recommendations if return | 9 |
| 10 | **WRITTEN english**  a. Spelling | 9 |
|  | b. Grammar | 9 |

Length of the paper:

|  | **Should be extended** | **Perfect** | **Should be shortened** |
| --- | --- | --- | --- |
| **ABSTRACT** |  |  | **X** |
| **PAPER** |  | **X** |  |

**This paper should be published as:**

| **Regular paper** | **X** |
| --- | --- |
| **Short paper** |  |
| **Commentary Paper** |  |

**This paper should be published as:**

| **TOP TOPIC IN THE JOURNAL** |  |
| --- | --- |
| **GOOD TOPIC IN THE JOURNAL** | **X** |
| **FAIR TOPIC IN THE JOURNAL** |  |

Types of bias:

| **Follow up bias** |  |
| --- | --- |
| **Exclusion bias** |  |
| **Analysis bias** |  |
| **Reporting bias** |  |
| **Assembly bias** |  |
| **Measurement bias** |  |
| **Detection bias** |  |
| **Selection (susceptibility) bias** |  |
| **Chronology bias** |  |
| **Channeling bias** |  |
| **Response bias** |  |

Types Conflicts of interests:

| **Between professionals** |  |
| --- | --- |
| **Competing loyalties** |  |
| **Relationship conflicts** |  |
| **Self-dealing** |  |
| **Structural conflicts** |  |
| **Communication** |  |
| **Data conflicts** |  |
| **personal** |  |
| **Gifts** |  |
| **Subject related** |  |
| **Interest conflicts** |  |
| **Value conflicts** |  |

| **Overall recommendation** | **YES** |
| --- | --- |
| Acceptable without any changes |  |
| Acceptable after minor modifications | **X** |
| Major revision: needs to be re-written and re-evaluated |  |
| Unacceptable |  |

**Questions to the authors:**

1. **You can use my suggestions**

**To the editor:**

**Dear Editor, thank you for giving me an opportunity to review this manuscript**

**This is an expert author**

**Please check the plagiarism**

**my final decision is acceptable after minor revision**

**Date: 25/8 / 2022**
