# Supplementary material for: Dynamic risk assessment of hospital oxygen supply system by HAZOP and intuitionistic fuzzy
Source: PLoS One. 2023 Feb 23;18(2):e0280918. doi: 10.1371/journal.pone.0280918 (PMC9949673; doi:10.1371/journal.pone.0280918)
Supplement: Attachment — Submitted filename: Response to comment.doc [file pone.0280918.s003.doc]

**Response to the Comments**

**1. We notice that your manuscript file was uploaded on August 17, 2022. Please can you upload the latest version of your revised manuscript as the main article file, ensuring that does not contain any tracked changes or highlighting. This will be used in the production process if your manuscript is accepted. Please follow this link for more information: http://blogs.PLOS.org/everyone/2011/05/10/how-to-submit-your-revised-manuscript/**

**A:** we performed according to the comment.

**2. Thank you for your attention to our requests. We have noted possible conflicting statements regarding the request for informed consent for the experts (occupational health and safety experts, artificial intelligence, medical equipment manager, hospital crisis manager, and device operators) involved in the study. Please could you clarify the following:**

**a.) Please confirm whether informed consent was obtained from this cohort of participants**

**b.) Please detail the type of informed consent (written or verbal). If verbal please confirm how verbal consent was documented.**

A: Our research was not a clinical study or medical interventions. Thus, we did not obtain informed consent from the experts. In this study, we asked the experts to give us information about the oxygen supply system that helped us in the risk assessment process.
